# Manual of Human Anatomy

**Published:** 1837-01

**Authors:** 


Art. II.—*Handbuch der Menschlichen Anatomie. Durchaus nach eigenen untersuchungen, und mit besonderer rücksicht auf das bedurfniss der Studirenden, der Practischen Aerzte und Wundärzte und der Gerichtsärzte verfasst von* C. F. T. Krause, m.d., Professor der Anatomie, &c. zu Hannover. I.-II. Abtheilung.—*Hannover,* 1833, 1836. 8vo. s. 632.

*Manual of Human Anatomy, compiled by* C. F. T. Krause, m.d., Professor of Anatomy, &c., *from his own Investigations, and with particular Reference to the Wants of the Student, the Physician, Surgeon, and Medical Jurist.* First and second Parts.—*Hanover,* 1833, 1836. 8vo. pp. 632.

Were this a common Manual of Anatomy, constructed on the usual model of such, by arranging the matter of preceding publications in a new form, without a single original addition, we would pass it over with the hundreds of foreign books which we omit to notice because they contain nothing new or of practical importance. But the work of Dr. Krause is of a different kind. Together with all that was previously known in the descriptive anatomy of the human body, expressed in the ancient form, and verified by the author's own investigations wherever any doubt existed, this little volume contains much that is placed in new points of view, and not a few things that are actually new. The most remarkable of the latter are the average weights and dimensions of all the organs and parts of the body, which are supplied by the author from his own observation and experiments. He informs us in his Preface that these weights and measurements were taken chiefly from the bodies of healthy individuals, who had come to their death suddenly, by design or accident. Fr. Meckel had previously done a good deal in this way, but the observations of the present author are much more complete. Although it is, of course, impossible to lay down any positive law as to the absolute size and gravity of the different organs, it cannot be denied that a general average can be drawn, any more than it can be doubted that the knowledge of such an average must prove very useful in many cases in determining the true condition of parts observed in subjects who have perished of disease. As an example of this mode of estimating the normal condition of organs, we give a part of the statements respecting some of the abdominal viscera, which are found frequently altered in their dimensions and weight by disease.

"*The Liver.* The length of the liver is from 10 to 12 inches;* its breadth, from its acute to its obtuse border, about 7 to 7¾ in., near its extremities only 5 in.; its greatest thickness, from the convex to the concave side, is nearer the obtuse than the acute border, and is about 2½ or 2¾ in., near the extremities it is considerably thinner. The absolute weight of the liver varies from 4½ to 6 lbs., the mean being about 5½ lbs. The specific gravity is from 1.0654 to 1.0853, giving an average of 1.0721; the solid contents range from 76¾ to 98⅓ cubic in., the average being 88." (P. 508.)

"*The Spleen.* The spleen measures from above below 5 to 5½, from the anterior to the posterior border, 3 to 4, from the outer to the inner surface, 1¼ to 1⅔ inches; its weight varies from 7 to 10½ oz., the average being 8½; the specific gravity is 1.0579 to 1.0625, the mean 1.0606; the solid contents 9¾ to 15 cubic in., the average 12⅓." (P. 519.)

"*The Pancreas.* The pancreas is 7 to 8 in. long; 2½ broad at the larger extremity, and ¾ thick; in the middle it is 1½ broad and ½ thick; the lesser extremity is only a little slenderer, and is often a little thicker than the middle portion. The weight is from 2¼ to 3½ oz.; the mean specific gravity, 1.0462; and the solid contents from 3⅓ to 5⅓ cub. in." (P. 517.)

"*The Kidney.* The length of the kidney is from 4 to 4½ in.; the breadth from 2 to 2⅓, and in the upper part 2⅔ in.; the thickness, from the anterior to the posterior surface, is from 1¼ to 1⅔ in. The weight is from 4 to 6 oz. The specific gravity from 1.0492 to 1.0555, the mean 1.0520; the solid contents from 5⅔ to 9½ cub. in., the average 7½." (P. 522.)

We think this work deserves translation, and would well repay the labour to an industrious student. It is printed in the Roman type, on good paper, and the whole getting-up is creditable to "the trade" of our sister-kingdom of Hanover.

* Almost every state in Germany has a different foot and inch measure. In scientific explanations, the *Rheinländishe* measure is generally used, and is, we presume, that adopted by Dr. Krause: in this the foot has twelve inches, and the inch twelve lines; the proportion of the Rhenish to the English foot being as 34 to 35.

